# New solid-state membrane and coated wire potentiometric sensors for the determination of Zn(II) ions based on nanoparticles

**DOI:** 10.1007/s00216-022-04359-y

**Published:** 2022-10-14

**Authors:** Mohamed A. F. Elmosallamy, Hassan A. Hashem, Fatma F. Abdalmoez

**Affiliations:** 1 Department of Chemistry, Faculty of Science, Zagazig University, Zagazig, 44519 Egypt; 2 Nano Materials Research Lab., Department of Physics, Faculty of Science, Zagazig University, Zagazig, 44519 Egypt

**Keywords:** ZnS nanoparticles, XRD, TEM, Solid-state membrane sensor, Coated wire sensor, Analytical applications

## Abstract

The first, novel solid-state membrane sensor for Zn(II) determination is developed based on ZnS nanoparticles. ZnS nanoparticles are synthesized by chemical co-precipitation and investigated via X-ray diffraction, transmission electron microscopy (TEM), Fourier transform infrared spectroscopy (FTIR) and impedance study. X-ray diffraction shows that the prepared ZnS nanoparticles have an average domain size of 5.72 nm, which is very close to the particle size obtained from TEM observations (6.30 nm). The ZnS nanoparticles are pressed into disks and examined as electroactive solid-state membrane. Solid-state membrane and coated wire sensors are fabricated. They display linear responses over concentration ranges of 1.0 × 10^−5^ to 1.0 × 10^−1^ mol L^−1^ Zn^2+^ ions with cationic slopes of 28.9±0.2 and 25.9±0.2 mV decade^−1^ for the solid-state membrane and coated wire sensors, respectively. The lower limits of detection are 2.86 × 10^−6^ and 4.60 × 10^−6^ mol L^−1^ Zn^2+^ ions for the solid-state membrane and coated wire sensors, respectively. The response time for the two sensors is instantaneous (1 s), and the useful lifetimes for the solid-state membrane and coated wire sensors are long (10 and 6 months, respectively). The solid-state membrane sensor is utilized for the quantification of Zn(II) ions in brass alloys and pharmaceutical preparations.

## Introduction

Zinc metal follows copper in the periodic table and has two *s* electrons outside the filled *d* orbital. It occurs widely in many minerals, but the main source is sphalerite (ZnFe)S. Zn reacts with non-oxidizing acids, releasing hydrogen and creating divalent ions. Zinc also dissolves in some strong bases, forming zincate ions (ZnO_2_^2−^). Moreover, zinc plays a very important biological role, as the Zn^2+^ ion is contained in many enzymes such as alkaline phosphatase, peptidase, dehydrogenase, both DNA and RNA polymerase, and phospholipase, in addition to its importance in carbohydrates, lipids, and protein metabolism in virtually all organisms [1]. Zn^2+^ ions play an important role in the formation and function of the immune system. They are necessary for normal growth and the healing of wounds and burns. On the other hand, excess zinc ions can be toxic and cause pollution to the environment [2]. Furthermore, zinc sulphide nanoparticles are notable as a significant inorganic semiconductor. However, the shape, the size, and the approaches for preparation are significant factors that determine the features of nanoparticles (NPs). The characteristics of nanoparticles can also be tuned for a particular application by designing their shape and size [3]. The characteristics of ZnS nanoparticles make it them attractive contender for spintronic optoelectronics and biomedical applications, such as biosensors and biocomposites [4].

In light of the above, the estimation of Zn^2+^ ions in the environment, medicine, etc., has become a significant area of interest. Many approaches are described for the determination of zinc(II) ions, such as UV–visible spectrometry [5], fluorimetry [6], atomic absorption spectrometry [7] and stripping voltammetry [8]. Most of these methods require expensive instruments, are time-consuming and necessitate pretreatment of the samples. As an alternative to these approaches, ion-selective membrane sensors are comparatively affordable, fast, and quite simple. Numerous PVC-based membrane sensors using different ionophores for the determination of Zn^2+^ ions have been described [9–28]. Solid-contact zinc(II) PVC membrane sensors have also been reported [29, 30]. An all-solid-state PVC membrane Zn^2+^ ion sensor has been fabricated [31]. In addition to this, carbon paste electrodes for zinc(II) have been reported [32–37]. A zinc(II) ion sensor based on NiO nanostructure doped with PVC membranes has also been reported [38].

In reviewing the literature, we could not find a reported solid-state membrane sensor for the estimation of Zn^2+^ ion. And unfortunately, there is no commercially available zinc(II) solid-state membrane sensor, since the ZnS material is hygroscopic. Herein we report the first, novel zinc(II) solid-state membrane sensor based on ZnS nanoparticles, and we also developed a Zn(II)-coated wire sensor. The former sensor is free from the troublesome features of the liquid/PVC membrane sensors such as leaching of the electroactive components, evaporation, and short life span. The developed sensors are considered unique sensors.

## Experimental

### Reagents and materials

All chemicals used were of high purity, and bi-distilled water was utilized. Zn (NO_3_)_2_.6H_2_O, ZnCl_2_, and sodium sulphide nonahydrate (Na_2_S.9H_2_O) were purchased from Merck & Co. (Germany). Sodium hydroxide was obtained from Alpha Chemika and butyl alcohol CH_3_(CH_2_)_3_OH from El-Nasr Pharmaceutical Chemical Company (Egypt). ZnS nanoparticles were prepared as described below. Dosage forms containing Zn^2+^ ions were obtained from local drugstores. Synthetic brass alloys were prepared in the laboratory.

### Preparation of zinc(II) sulphide nanoparticles

ZnS NPs were produced by chemical co-precipitation. We synthesized the ZnS NPs as described previously [39]. Briefly, ZnS nanoparticles were prepared by dissolving 17.04 g of ZnCl_2_ and 23.26 g of Na_2_S.9H_2_O in 500 mL bi-distilled water. ZnS was precipitated by slowly adding excess aqueous solution of NaOH (1.0 mol/L) to the mixture of ZnCl_2_ and Na_2_S.9H_2_O aqueous solution under continuous stirring during the reaction. The value of the pH of the solution was 13. The precipitate was washed with bi-distilled water and then dried in an oven for 5 h at 80 °C; finally, a whitish powder of ZnS nanoparticles was obtained.

### Preparation of the sensors

**Fig. 1:**
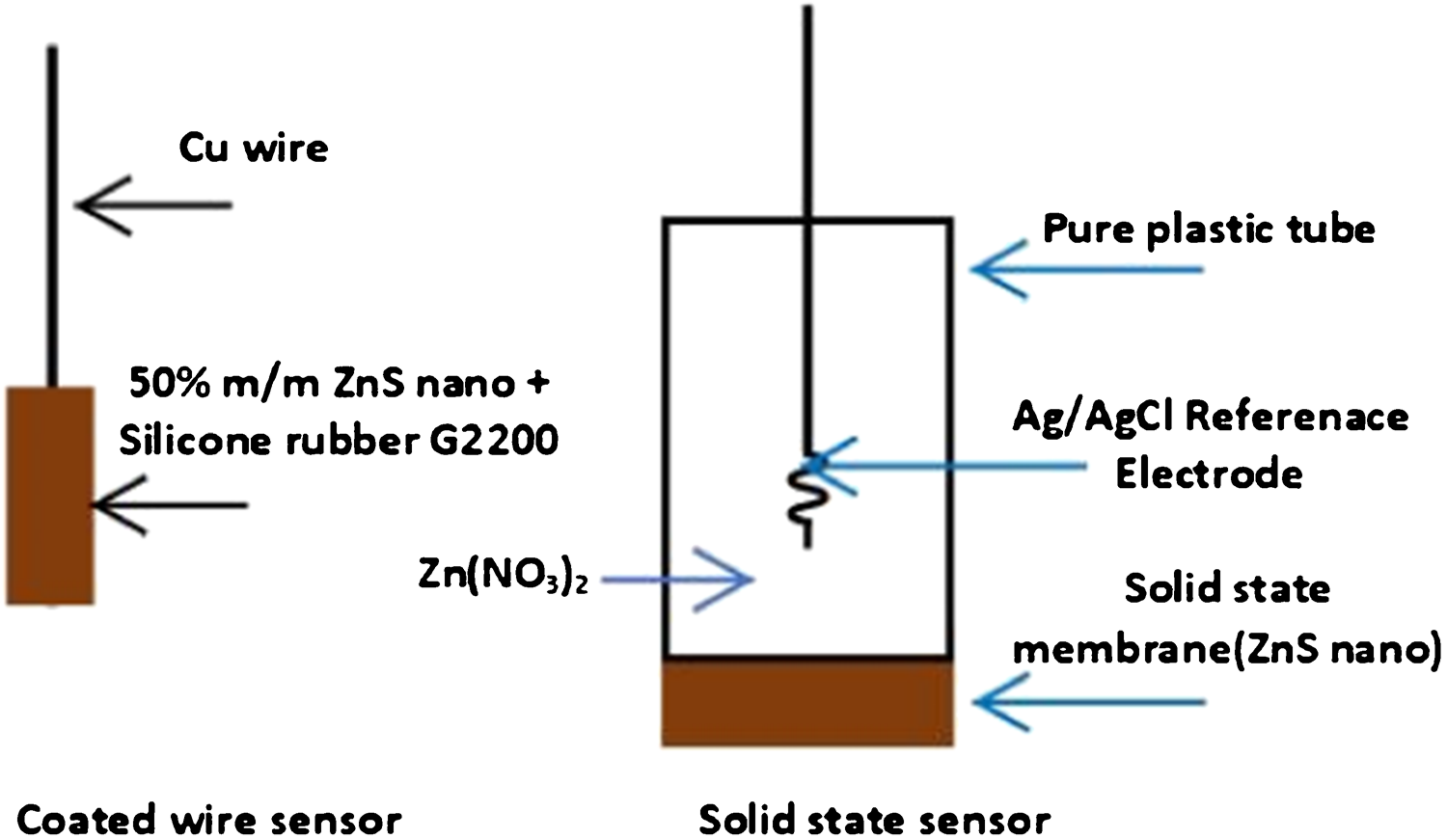
Schematic diagrams for solid-state membrane and coated wire sensors

Solid-state membrane (Fig. 1) was prepared by pressing 1.0 g of finely powdered ZnS nanoparticles using pressure of 10 tons to yield a disk of 1.3 cm in diameter and 2.5 mm in thickness. The obtained disk was sealed with PVC glue to the end of pure plastic tube of the same diameter, as a first adhesive, and then pure epoxy adhesive was applied to obtain perfect sealing and prevent any possible leak. The internal reference electrode was Ag-AgCl wire immersed in 1.0 x 10^−2^ mol L^−1^ Zn (NO_3_)_2_ internal filling solution. The developed sensor was conditioned by soaking in a 1.0 x10^−2^ mol L^−1^ zinc(II) nitrate solution for 24 h before use, and stayed in the same solution when not in use.

Coated wire sensor (Fig. 1) was prepared as follows: a finely powdered ZnS nanoparticles was mixed well with clear silicone rubber in the ratio of 50% (mass/mass) to obtain a homogeneous mixture. Tip of a clean, slightly polished copper wire of 8.0 mm in length and 1.0 mm diameter was dipped several times in the mixture to coat the tip and get a bead, then left in air for one day to get complete dryness before use. The sensor was stored in a 1.0 x10^−2^ mol L^−1^ Zn (NO_3_)_2_ solution.

### Apparatus

Potentiometric measurements were performed at room temperature with a PTI-15 digital pH meter using the proposed sensors. The reference electrode was an EIL-type RJ 23 calomel electrode. A glass Ag-AgCl combination electrode (Consort, 5210 B BB5) was used for measuring the pH. Atomic absorption measurements were carried out using a Thermo Scientific ICE 3000 Series atomic absorption spectrometer (UK). X-ray diffraction (XRD) spectra were recorded utilizing a Philips X'Pert MPD diffractometer (PANalytical, Netherlands). Monochromatized Cu Kα (λ=0.1541 nm) was utilized as the X-ray source and worked at 40 kV and 20 mA. Diffraction patterns were recorded over a 2θ range of 10° to 80° in steps of 0.02°. The infrared spectroscopic absorption data in the range of 4000–400 cm^−1^ were obtained with Fourier transform infrared (FTIR) spectroscopy using a Bruker ALPHA FTIR spectrometer with an attenuated total reflectance accessory (FTIR-ATR). The morphology of the materials was investigated using a JEOL JEM-1230 transmission electron microscope (TEM). The complex impedance for the ZnS nanoparticles in a frequency range from 42 Hz up to 5 MHz was measured using an LCR tester (Hioki 3532-50 HiTester).

### Sensor calibration

The calibration of the sensors was performed by dipping the solid-state membrane or coated wire sensor and the calomel reference electrode in 50-mL beakers containing 25 mL of the standard 1.0 x 10^−6^ to 1.0 x 10^−1^ mol L^−1^ of Zn^2+^ ion solutions. The readings of the potential were recorded when became constant, and plotted as a function of the logarithm of the Zn ^2+^ ion concentrations. On the other hand, we measured the pH of the standard solutions simultaneously with the potential readings and found that the pH values lay at the working pH range of the sensors. Furthermore, Zn(NO_3_)_2_ is a strong electrolyte; therefore, there is no impact for the ionic strength on the sensors response. The calibration graph has been utilized for the estimation of the unknown Zn^2+^ ion concentrations dealing with only solid-state membrane sensor. This sensor is better than the coated wire sensor in sensitivity and has a longer life span, as we will see in the results and discussion section.

### Sensor selectivity

The potentiometric selectivity coefficients $\left({\mathrm{k}}_{\mathrm{Zn},\mathrm{B}}^{\mathrm{pot}}\right)$ were estimated utilizing the separate solution method [40–42]. Calibration of the solid-state membrane sensor or the coated wire sensor was performed using 25 mL of 1.0 ×10^−6^ to 1.0 ×10^−1^ mol L^−1^ of Zn^2+^ ions, and the potential value corresponding to the concentration of 1.0 ×10^−2^ mol L^−1^ was then determined as E_Zn_. For each interferent, the potential value corresponding to the concentration of 1.0 × 10^−2^ mol L^−1^ was determined as E_B_. The values of (${\mathrm{k}}_{\mathrm{Zn},\mathrm{B}}^{\mathrm{pot}}$) were obtained from Eq. [40]:

$$\log \left({\mathrm{k}}_{\mathrm{Z}\mathrm{n},\mathrm{B}}^{\mathrm{pot}}\right)=\frac{E_{B-}{E}_{Zn}}{S}+\left(1-\frac{{\mathrm{Z}}_{\mathrm{Z}\mathrm{n}}}{Z_B}\right)\log \left({a}_{Zn}\right)$$

where E_Zn_ and E_B_ are the potential values of the Zn(II) and interferent, respectively. S is the slope of the calibration plot, a_Zn_ is the activity of Zn(II), and Z_Zn_ and Z_B_ are the charges of Zn(II) and the interferent, respectively.

### Determination of Zn^2+^ ions in pharmaceutical preparations

The contents of five tablets of zinc origin drug or two capsules of solvazinc or four capsules of octazinc were finely powdered. A portion of the powder equivalent to one tablet or one capsule was dissolved in 25 mL of bi-distilled water and filtered into a 50-mL volumetric flask. The solutions were completed to the mark. The content was determined potentiometrically via a calibration graph and atomic absorption spectrometry (AAS).

### Determination of Zn^2+^ ions in brass alloys

Synthetic brass alloy 1 (60% Cu^2+^ + 40% Zn ^2+^) was prepared by mixing of 30 mL standard of 10^−2^ mol L^−1^ Cu SO_4_ with 20 mL of 10^−2^ mol L^−1^ Zn (NO_3_)_2_ into a 100-mL volumetric flask and completed with bi-distilled water to the mark. Synthetic brass alloy 2 (55% Cu^2+^ + 45% Zn^2+^) was prepared by mixing of standard 27.5 mL of 10^−2^ mol L^−1^ Cu SO_4_ with 22.5 mL of 10^−2^ mol L^−1^ Zn (NO_3_)_2_ and completed as previous. The contents of the Zn^2+^ ions were assayed potentiometrically, and using AA method.

## Results and discussion

ZnS nanomaterial was prepared, pressed into a pellet, and examined as an electroactive solid-state membrane for the Zn(II) sensor, and it was also examined as an electroactive material for the coated wire sensor of Zn^2+^ ions. ZnS nanomaterial is insoluble in water, not hygroscopic, in contrast to the non-nanomaterial ZnS.

## X-ray diffraction of ZnS nanoparticles

**Fig. 2:**
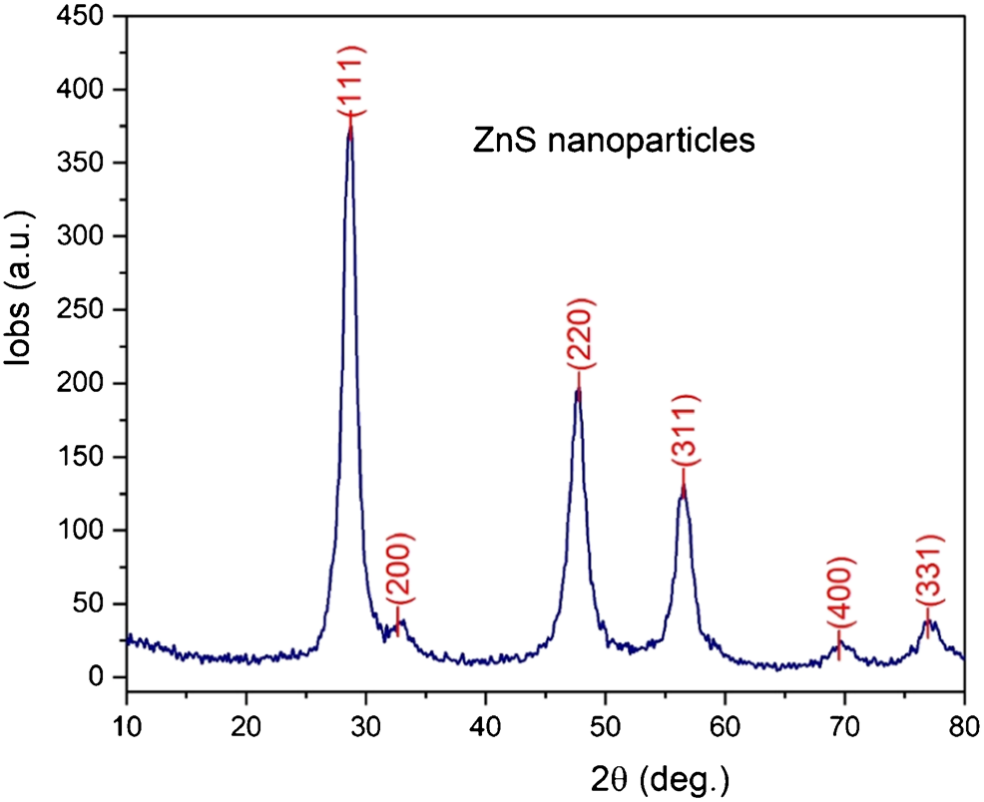
XRD spectra of the synthesized ZnS nanoparticles

X-ray diffraction spectrum (Fig. 2) showed that ZnS nanoparticles formed by cubic symmetry (Pm-3m space group) with lattice constant a=5.4112(9) Å and volume V=158.45(4) Å^3^. The peaks were indexed to (111), (200), (220), (311), (400) and (331), which matches very well with the International Centre for Diffraction Data (ICDD) reference card no. 04-004-3804 and the cubic zinc blend structure [43].

**Fig. 3:**
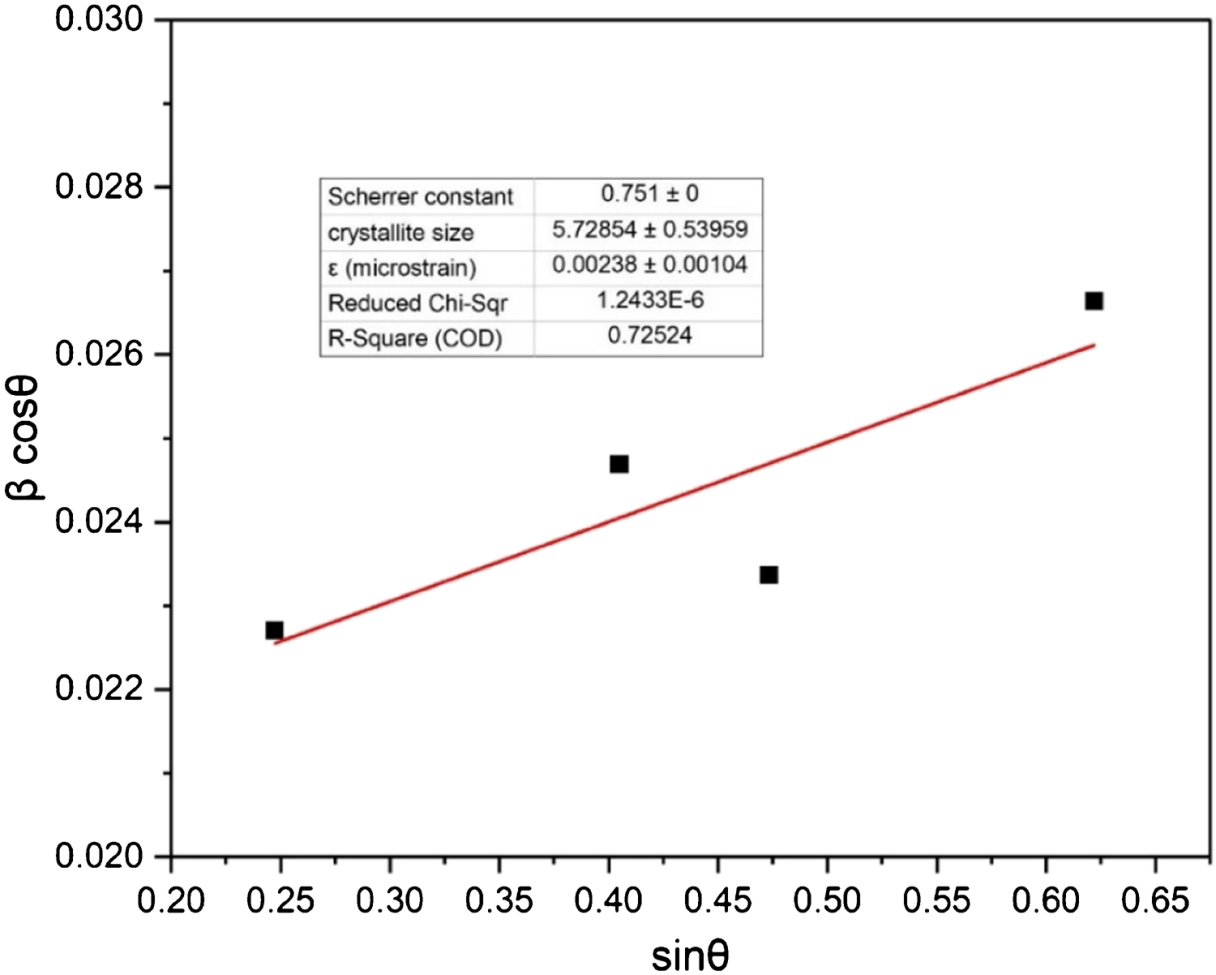
Williamson–Hall plot of the synthesized ZnS nanoparticles

The average crystallite size and the strain induced in the lattice were estimated with Williamson–Hall (W-H) plots using the following equation [44–46]:

$$\beta \cos \theta =4\varepsilon \sin \theta +\frac{K\lambda}{D}$$

where D is the mean crystallite size, K is constant shape factor with a value of 0.751 in spherical nanoparticles, λ is the wavelength of Cu Kα radiation (λ = 1.5406 Å), β is the full width at half maximum (FWHM), θ is the Bragg angle and ε is induced strain. The size and strain induced in the lattice was calculated by plotting β cosθ along the y-axis and sinθ along the x-axis. The intercept indicates that factor Kλ/D is the intercept and 4ε is the slope of the line. Hence, from the intercept, one can obtain the average crystallite size, whereas from the slope of the line, the induced strain can be calculated. W-H plots for ZnS are shown in Fig. 3. The estimated average crystallite size of ZnS and microstrain are 5.72±0.54 nm and 0.00238±0.00104, respectively [47].

### Transmission electron microscopy (TEM)

**Fig. 4:**
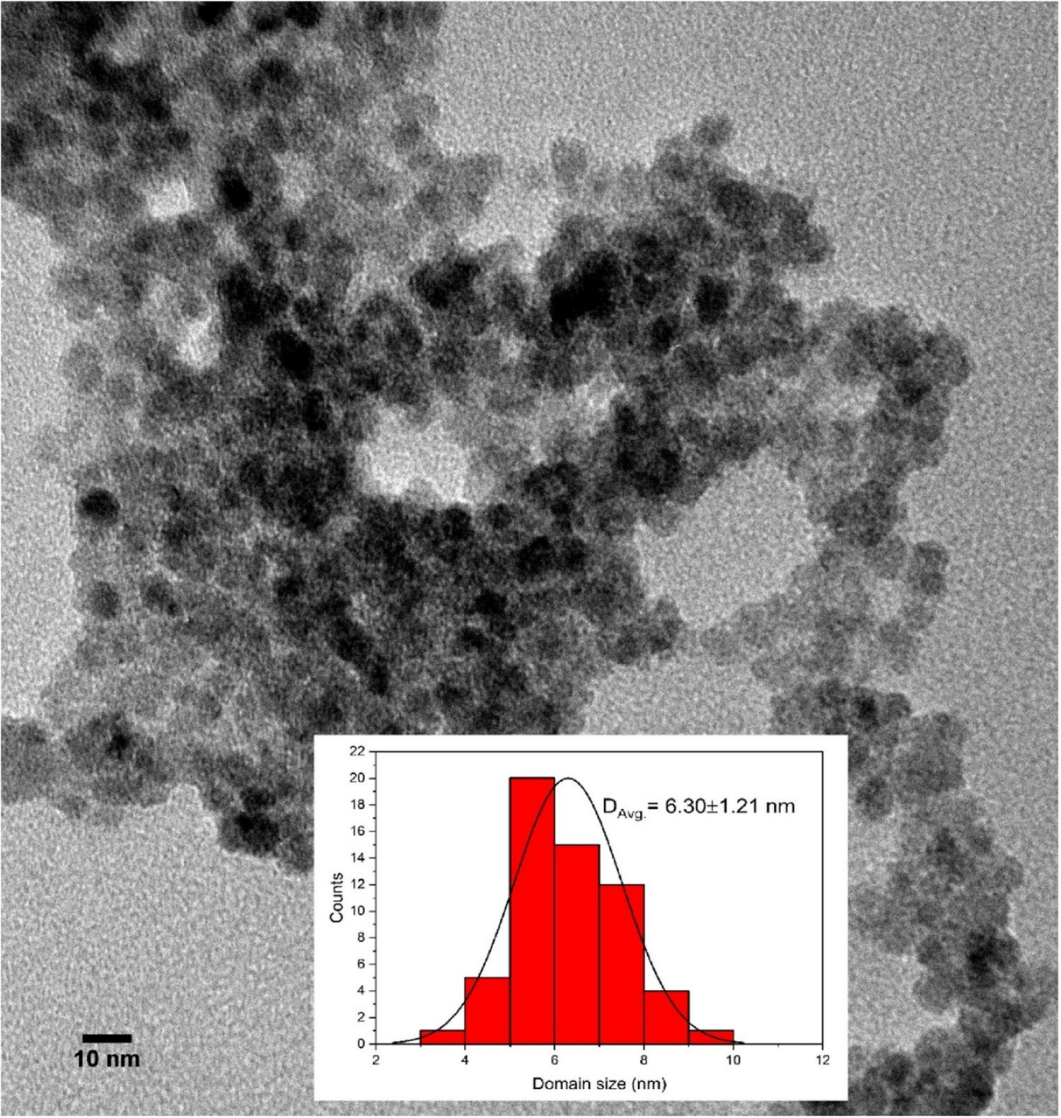
TEM image of ZnS nanoparticles (Inset: particle size distribution)

TEM image of ZnS nanoparticles was depicted in Fig. 4. The TEM image of ZnS sample showed the presence of large number of nearly spherical ZnS nanoparticles with distinct grain boundaries. The average particle size is found to be 6.3 nm which is nearly matched with the particle size obtained from XRD observations[46].

### Fourier transform infrared spectroscopy (FTIR)

**Fig. 5:**
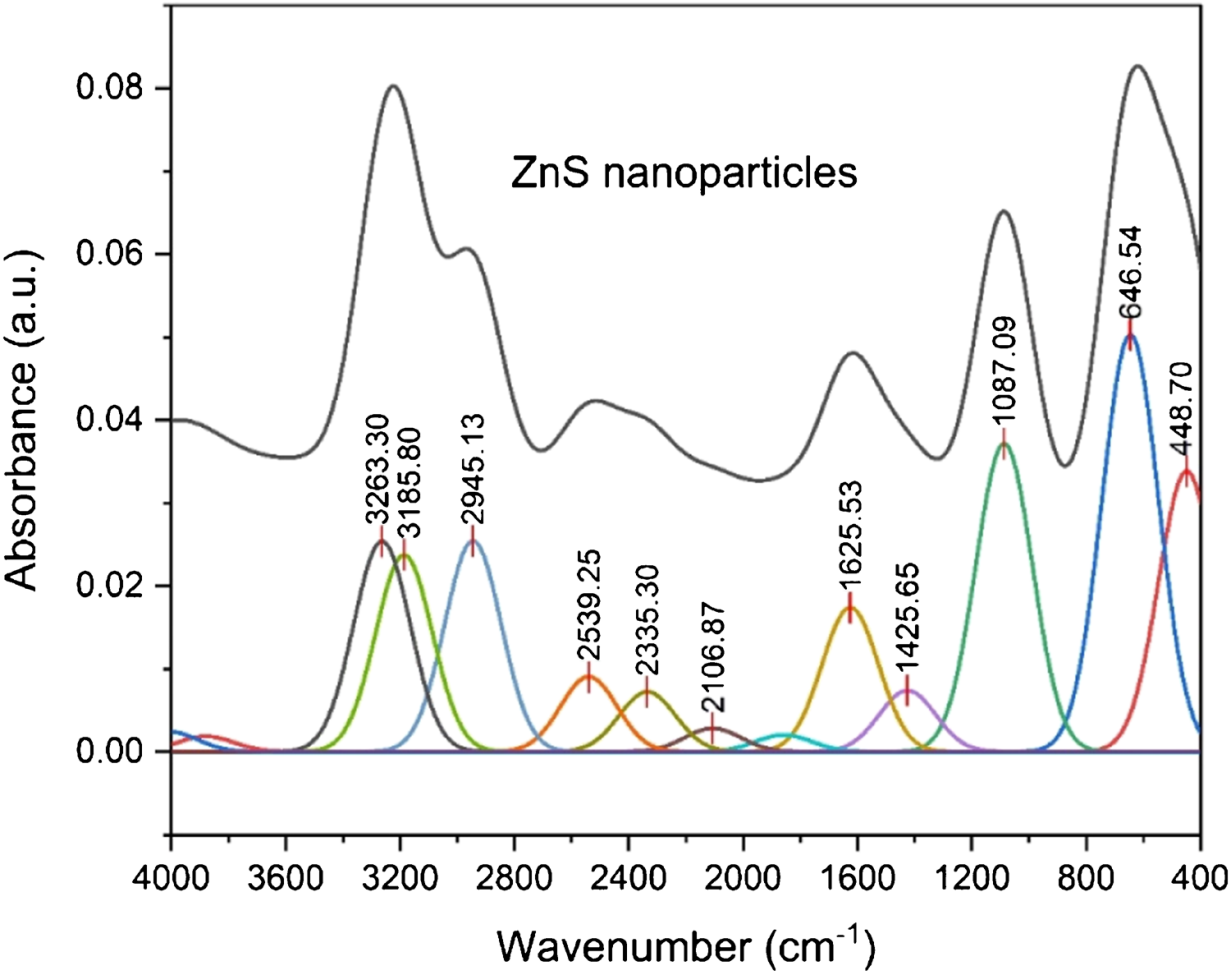
FTIR deconvolution spectra of ZnS nanoparticles

FTIR spectrum of zinc sulphide NPs is given in Fig. 5. The spectrum showed two strong to medium broad bands observed in the region at 3300–3200 cm^−1^, which may be attributed to the stretching motion of the O–H bond (*v*(*OH*). This band may have originated from water adsorbed on the ZnS surface. Two medium absorption bands lying in the region 2540–2300 cm^−1^ may be derived from microstructure formation of ZnS NPs [43]. The medium broad band which appeared at 1625 cm^−1^ is composed of two overlapped peaks corresponding to two motions: the bending motion *δ*(H_2_O) + the stretching motion, *v*(C=O) group arising from atmospheric CO_2_ adsorbed on the ZnS surface [48, 49]. Finally, the spectrum showed three bands at 1087, 646 and 448 cm^−1^, which may be attributed to the resonance interaction between vibrational modes of sulphide ions in ZnS crystal [50].

### Impedance study

**Fig. 6:**
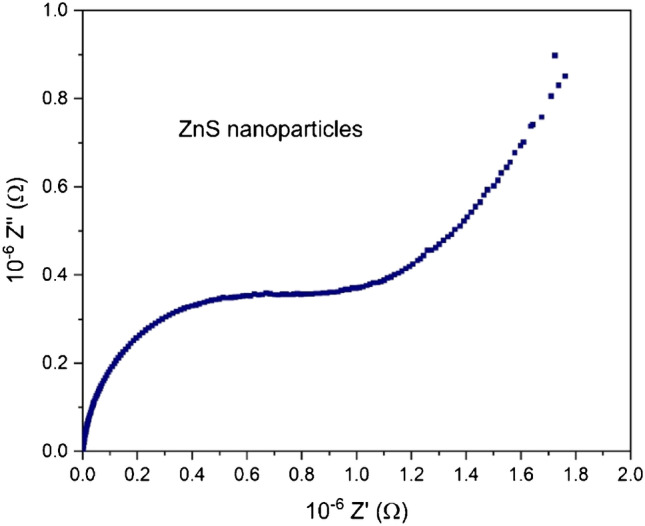
Plot of the imaginary Z″ (ω) versus real Z′ (ω) part of the complex impedance at 310 K

Impedance spectrum analysis is a sensitive and robust pragmatic method which makes it possible to plainly comprehend the connection between electrical properties and microstructure of ZnS nanoparticles, grains, and grain limits impacts. The variation in impedance Z″ with the real part Z′ of ZnS nanoparticles is represented in the Nyquist chart at T=310 K as depicted in Fig. 6. The examination of the dielectric unwinding identified with the grain limits for T=310 K shows that conduction is constrained by grain limits, which clarify the presence of a single semicircle in the Nyquist chart.

### Performance characteristics of the sensors

**Fig. 7:**
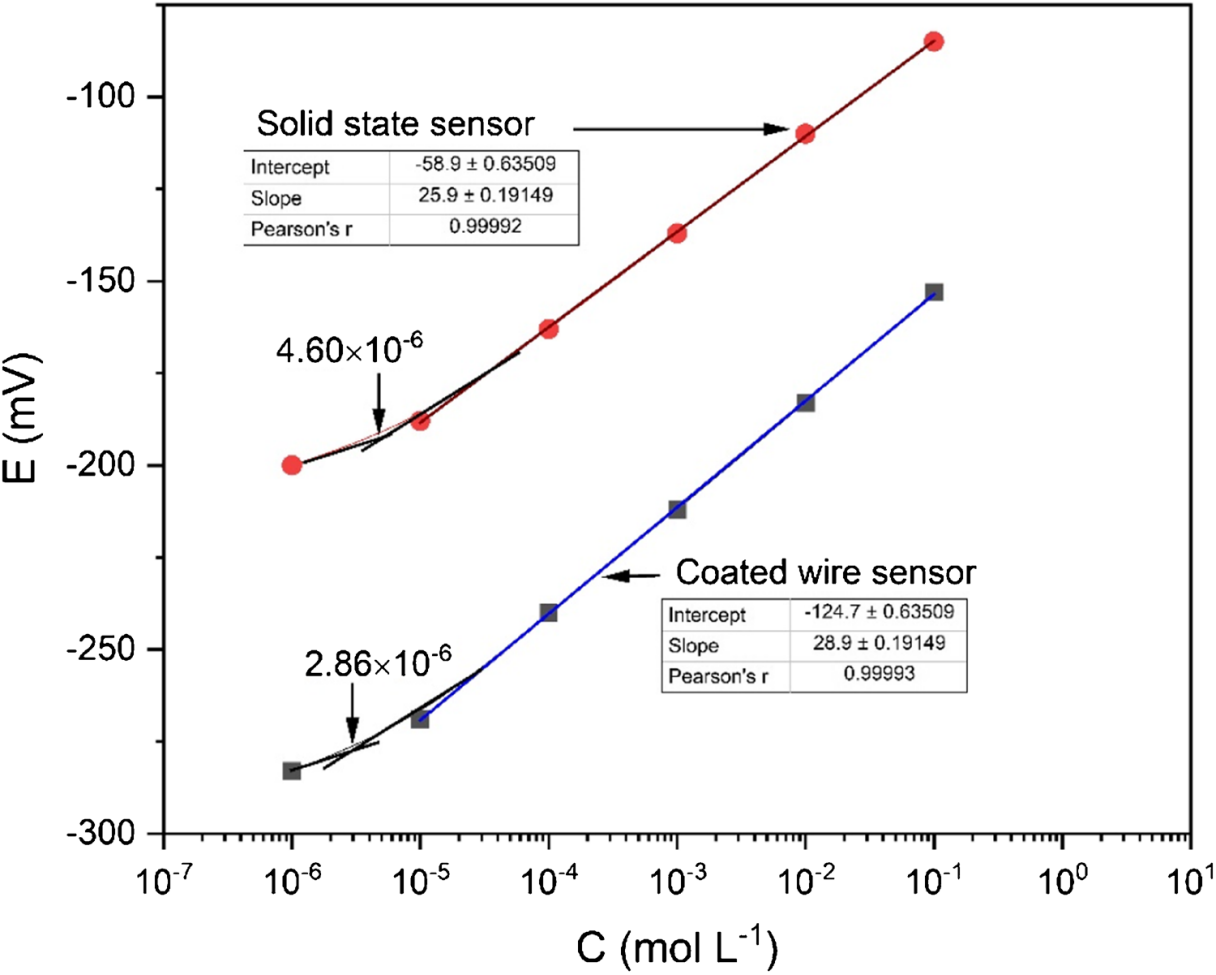
Potentiometric responses of the solid-state membrane and coated wire sensors

The two sensors were constructed as illustrated in an experimental part, and an electrochemical evaluation of the two sensors was done according to the IUPAC recommendations [51]. They showed linear response over the concentration ranges of 1.0 ×10^−5^ to 1.0 ×10^−1^ mol L^−1^ Zn^2+^ ions with cationic slopes of 28.9 ±0.2 and 25.9 ± 0.2 mV decade^−1^ for the solid-state membrane and coated wire sensors, respectively (Fig. 7) and (Table 1). The solid-state membrane sensor displays a Nernstian slope whereas the coated wire sensor has a sub-Nernstian slope, which may be because in the case of the coated wire sensor, a thin water layer formed between the metal and the electroactive material, causing high ohmic resistance that reduces the response. Therefore, the sensor has sub-Nernstian behavior. But in the case of the solid-state membrane sensor, there is a direct contact between the membrane and the sample. The lower limits of detection were 2.86 × 10^−6^ and 4.6 × 10^−6^ mol L^−1^ Zn^2+^ ions for the solid-state membrane and coated wire sensors, respectively (the lower limit of detection may be taken as the concentration of Zn(II) ions at the point of intersection of the extrapolated midrange and the final low concentration level segments of the calibration plot [51]). The response time for the two sensors was instantaneous (1 s), and the useful lifetimes for the solid-state membrane and coated wire sensors were quite long, 10 and 6 months, respectively. After that, the calibration slope decreased gradually.

**Table 1:** Potentiometric response characteristics of the solid-state membrane and coated wire sensors

Parameter	Solid-state membrane sensor	Coated wire sensor
Slope (mV) decade^−1^	28.9±0.2	25.9±0.2
Intercept (mV)	−124.7±0.6	−58.9±0.6
Correlation coefficient (r)	0.99993	0.99992
Lower limit of linear range (mol L^−1^)	1.0×10^−5^	1.0×10^−5^
Lower limit of detection (mol L^−1^)	2.86×10^−6^	4.6×10^−6^
Working pH range for 1.0×10^−3^ (mol L^−1^)	3.5–8	3.7–7.3
Response time (s) for 1.0×10^−3^ (mol L^−1^)	1	1
Life span (months)	10	6
Accuracy (%)	99.9± 0.4	98.7± 0.5
Repeatability, CV_w_ (%)	0.9	1.3
Between-day variability, CV_b_ (%)	1.2	1.4

## Effect of the pH

**Fig. 8:**
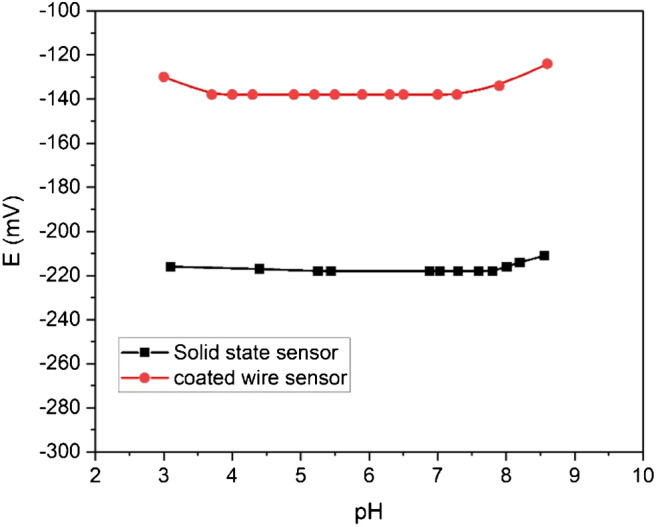
pH profile of solid-state membrane and coated wire sensors for 10^−3^ mol/L Zn(NO_3_)_2_

To obtain the useful pH range for the two potentiometric sensors (Fig. 8), the influence of the change in pH on the potential was studied using 10^−3^ mol L^−1^ of Zn^2+^ ion solution over the pH range of 2–10. The pH was tested using dilute HCl acid and/or sodium hydroxide solution. From the pH-potential relationship, it is evident that the pH values were constant over the pH range of 3.5–8 and 3.7–7.3 for the solid-state membrane and coated wire sensors, respectively (Table 1). At a pH value lower than 3.5 or 3.7 for the solid-state membrane or coated wire sensor, an increase in potential readings was observed, which may be due to the interference of H_3_O^+^ ions. At pH values higher than 8 for the solid-state membrane and 7.3 for the coated wire sensors, an increase in potential also occurred, probably due to the interferences by OH^−^ ions.

## Effect of the foreign ions

To show the selectivity of the two sensors, their potentiometric response was examined in the presence of numerous interferents, including NH_4_^+^ ion, six non-transition metals and seven transition metals. The potentiometric selectivity coefficients (${k}_{Zn,B}^{Pot}$) were calculated using the separate solution method [40]. The results are listed in Table 2, and they reveal that the two sensors had very high selectivity for Zn(II) ions over the NH_4_^+^ and 13 common transition and non-transition metal ions. In other words, the data showed that the two sensors have very high selectivity towards Zn^2+^ ions over monovalent ions; NH_4_^+^, Na^+^, and K^+^ (${k}_{Zn,B}^{Pot}$~ 2.4×10^−4)^ and high selectivity for Zn^2+^ ions over the listed of bi- and trivalent metal ions (${k}_{Zn,B}^{Pot}$ ~ 4.2× 10^−3^).

**Table 2:** Potentiometric selectivity coefficients (${k}_{Zn,B}^{Pot}$ ) of the solid-state membrane and coated wire sensors

Interferent, B	Solid-state membrane sensor	Coated wire sensor
Na^+^	3.31×10^−4^	1.40×10^−4^
K^+^	1.70×10^−4^	3.70×10^−4^
NH_4_^+^	2.24×10^−4^	8.80×10^−4^
Ca^2+^	6.90×10^−3^	2.70×10^−3^
Mg^2+^	4.60×10^−3^	3.60×10^−3^
Al^3+^	3.80×10^−3^	1.20×10^−3^
Pb^2+^	8.70×10^−3^	3.20×10^−3^
Mn^2+^	2.70×10^−3^	4.20×10^−3^
Co^2+^	4.10×10^−3^	2.00×10^−3^
Cu^2+^	3.40×10^−3^	8.60×10^−3^
Cd^2+^	8.30×10^−3^	1.00×10^−3^
Ni^2+^	6.80×10^−3^	2.50×10^−3^
Cr^3+^	2.40×10^−3^	2.6×10^−3^

## The analytical applications

The validity of the developed solid-state membrane and coated wire sensor methods for assaying of Zn(II) ions was assessed by calculating the linear range, sensitivity (slope), linearity (correlation coefficient), lower limit of detection (LOD), accuracy (recovery), precision or repeatability (CV_w_), between-day variability (CV_b_) [52] (Table 1). The results on five batches (five determinations each) with standard 6.54 μg mL^−1^ to 6.54 mg mL^−1^ of Zn(II) ion solutions using the calibration graph method gave average recoveries of 99.9% and 98.7%, with mean relative standard deviations of ± 0.4% and ± 0.5%, for solid-state membrane and coated wire sensors, respectively (Table 1).

Zn(II) ions were also determined in brass alloys and different pharmaceutical preparations using the proposed solid-state membrane sensor and atomic absorption spectrometry methods. The results showed a good correlation between the two techniques, where the average recoveries of the sensor method were 99.90% and 99.85% for the alloys and drugs, respectively, whereas the average recoveries with the AAS technique were 100.72% and 99.82% for the alloys and drugs, respectively (Tables 3, 4). The *F*-test revealed that there was no significant difference in the precision between the two methods, and the Student *t*-test calculated at the 95% confidence level showed that the calculated value is less than the tabulated one, indicating the accuracy of the developed sensor method.

**Table 3:** Determination of zinc(II) ions in brass alloys using solid-state membrane sensor and AAS

Brass alloy	Taken (ppm)	Found (ppm)		Recovery^a^ ± RSD %	
		Calibration graph method	AAS	Calibration graph method	AAS
Sample 1	130.80	130.76	133.99	99.97 ± 0.53	102.44 ± 0.41
Sample 2	147.15	146.90	145.68	99.83 ± 0.62	99.00 ± 0.32

^a^Average of six measurements

**Table 4:** Determination of zinc(II) ions in drug samples using solid-state membrane sensor and AAS

Drug name and source	Nominal content	Taken (ppm)	Found (ppm)		Recovery^a^ ± RSD%	
			Calibration graph method	AAS	Calibration graph method	AAS
Octozinc (October Pharma, Egypt)	25 mg/capsule	500	500.80	508.7	100.16 ± 83	101.6 ± 72
Zinc origin (EGPI, Egypt)	20 mg/tablet	400	398.80	393.3	99.70 ± 64	98.31 ± 81
Solvazinc (Al-Esraa Pharmaceuticals, Egypt)	50 mg/capsule	1000	996.88	995.5	99.69 ± 91	99.55 ± 53

^a^Average of five measurements.

## A comparison between the developed sensors and the reported sensors

To further study the potentiometric response characteristics of the solid-state membrane and coated wire sensors, they were compared with different sensors reported in the literature (Table 5). The results showed that the developed sensors displayed a longer life span and shorter response time than all the reported sensors. The solid-state membrane sensor also exhibited the Nernstian slope better than most of the reported sensors.

**Table 5:** Comparison of the developed Zn(II) sensors with different reported sensors in the literature

Sensor type	Slope mV/decade	Linear range mol L^−1^	Detection limit mol L^−1^	Working pH range	Response time (s)	Life span (months)	Reference
PVC based on acyclic arylamine ionophores	30.00	*	1.3×10^−6^	3.6–9.3	15	4	[9]
PVC based on neutral ion carrier	27.12	1.0×10^−5^ to 1.0×10^−1^	8.0×10^−6^	4.4–8.0	5	2	[10]
PVC based on 12-crown-4	20.50	7.1×10^−5^ to 1.0×10^−1^	1.41×10^−5^	4.0–7.0	<10	3	[14]
PVC based on tiophene-2-aldehyde semicarbazone ligand	29.20	1.0×10^−6^ to 1.0×10^−1^	*	2.4–6.7	15	2	[27]
All-solid-state sensor based on PVC	28.00	1.0×10^−5^ to 1.0×10^−1^	1.17×10^−6^	4.0–11.0	5	*	[31]
Carbon paste based on modified core/shell Fe_2_O_3_@ SiO_2_ nanoparticles	29.45	2.5×10^−6^ to 1.0×10^−1^	1.0×10^−6^	4.0–6.0	14	*	[34]
Coated wire based on PVC	*	1.0×10^−5^ to 1.0×10^−1^	*	1.5–6.0	*	*	[53]
Solid-state membrane sensor based on ZnS nanoparticles	28.9	1.0×10^−5^ to 1.0×10^−1^	2.86×10^−6^	3.5–8.0	1	10	This work
Coated wire based on silicone rubber and ZnS nanoparticles	25.9	1.0×10^−5^ to 1.0×10^−1^	4.60×10^−6^	3.6–7.3	1	6	This work

* Not mentioned

## Conclusion

The first, novel solid-state membrane sensor for Zn(II) estimation was developed, based on ZnS nanoparticles. A new coated wire sensor was fabricated based on the same nanoparticles. Moreover, the former sensor was free from the troublesome features of liquid/PVC membrane sensors such as leaching of the electroactive components, evaporation, and short life span. The developed sensors are considered to be far improved over the reported sensors. Also, the solid-state membrane sensor has a long life span (10 months) and was easy to design. The two sensors are simple, sensitive, and cost-effective.

## Declarations

The submitted work has originality and novelty and has not been published elsewhere.

The authors declare that this research involved no human participants or animals.
